# Effects of Interaction between *SLC35F3* and Carbohydrate Intake on the Incidence of Metabolic Syndrome in Korean Middle-Aged Adults

**DOI:** 10.3390/nu15020469

**Published:** 2023-01-16

**Authors:** Haeun Park, Dayeon Shin

**Affiliations:** Department of Food and Nutrition, Inha University, Incheon 22212, Republic of Korea

**Keywords:** *SLC35F3*, metabolic syndrome, carbohydrate, diet–gene interaction, single-nucleotide polymorphism (SNP)

## Abstract

Solute carrier family 35 member F3 (*SLC35F3*) mediates intracellular thiamine transport, which is crucial for carbohydrate metabolism as thiamine is required for key pathways such as glycolysis and the tricarboxylic acid cycle. This study aimed to investigate the impact of the interaction between *SLC35F3* and dietary carbohydrate intake on the incidence of metabolic syndrome (MetS). The study included 3923 Korean adults over 40 years of age from the Korean Genome and Epidemiology Study. The association between dietary carbohydrate intake, *SLC35F3* rs10910387 genotypes, and MetS incidence was studied using multivariable Cox proportional hazard models. Over an average of 8.5 years of follow-ups, we documented 1471 MetS cases. MetS incidence was 1.88 times greater in men with the TT genotype and the highest carbohydrate intake than in those with the CC genotype and lowest carbohydrate intake (Hazard Ratio (HR) 1.88, 95% confidence interval (CI) 1.03–3.41). MetS incidence were 2.22 and 2.53 times higher in women with the TT genotype and carbohydrate intake tertile 2 and 3, respectively, than those with the CC genotype and carbohydrate intake tertile 1 (HR 2.22, 95% CI 1.12–4.42; HR 2.53, 95% CI 1.38–4.61). In summary, we report a novel interaction between *SLC35F3* rs10910387 genotypes and dietary carbohydrate intake on MetS in Koreans.

## 1. Introduction

The “Syndrome X” cluster of clinical features include hypertension, dyslipidemia, abdominal obesity, diabetes, and insulin resistance [1]. Syndrome X, renamed metabolic syndrome (MetS), is defined as condition characterized by three or more of the following five conditions: abdominal obesity, hypertriglyceridemia, hypertension, diabetes, and low-density lipoprotein (HDL) cholesterolemia [2,3].

The prevalence of MetS has increased in Korea over the last 12 years, from 21.5% in 2007 to 22.9% in 2018 [4]. It increased considerably among men, from 22.5% in 2007 to 27.9% in 2018 [4]. Cardiovascular and cerebrovascular diseases are the primary causes of death in Korea, and MetS further increases the prevalence and mortality rate of these disorders [5]. Furthermore, because MetS is primarily caused by insulin resistance, it may also increase the prevalence of type 2 diabetes. Therefore, addressing MetS from a preventive perspective is critical as this condition can increase the prevalence of other chronic diseases.

MetS is caused by complex interactions between environmental, metabolic, and genetic factors [6]. Dietary factors, along with environmental factors, contribute to the genetic susceptibility to MetS [7]. A high-carbohydrate diet is associated with an elevated risk of MetS, especially as rice is a core food in the Korean diet [8,9]. Studies performed in Korean adults aged between 40 and 69 years showed that a carbohydrate-derived energy higher than 75.2% increased the risk of MetS by 1.34 times compared to a carbohydrate-derived energy lower than 67% and was particularly associated with abdominal obesity (Odds ratio (OR) 1.34; 95% confidence interval (CI) 1.08–1.66) [10]. In Korean adults aged between 20 and 64 years, people who consumed more than 70.1% carbohydrate-derived energy increased the risk of MetS by 1.35 times in men and 1.27 times in women than those who consumed less than 61% of carbohydrates, regardless of other nutrients (OR 1.35, 95% CI 1.08–1.68; OR 1.27, 95% CI 1.03–1.56, respectively) [8]. Men aged 30-65 in the quintile 5 group with the highest carbohydrate intake were at 1.46 times higher risk of MetS than the lowest quintile group; moreover, women who consumed the highest in the quintiles of white rice and refined carbohydrates had a 1.72- and 1.74-fold higher risk of MetS, respectively, in comparison to those who consumed the lowest quintile of white rice and refined carbohydrates (OR 1.46, 95% CI 1.07–2.01; OR 1.72, 95% CI 1.24–2.40; OR 1.74, 95% CI 1.23–2.48, respectively) [9]. Therefore, these results show that high carbohydrate intake in Koreans increases the risk of MetS.

A genome-wide association study (GWAS), which has made it possible to study complex diseases affected by various genetic or environmental factors due to the development of human genetic projects and microarray technology, revealed the association between solute carrier family 35 member F3 (*SLC35F3*) and MetS risk [11]. *SLC35F3* encodes a protein that facilitates intracellular thiamine migration. [12]. This gene is found on chromosome 1q42.2 and contains nine exons that encode a protein with 421 amino acids [13]. Korean men with the AT and AA genotypes of *SLC35F3* rs12135117 had a 0.86-fold lower risk of hypertension than those with the TT genotype (OR 0.86, 95% CI 0.74–1.00); furthermore, Korean women with the TC genotype of rs10910387 had a 1.17-fold higher risk of hypertension than those with the TT genotype (OR 1.17, 95% CI 1.00–1.37) [14]. In Chinese patients with hypertension, the CG and GG genotypes of *SLC35F3* rs34032258 were associated with elevated diastolic blood pressure levels [13]. Additionally, rs6699737 was associated with visceral fat and fasting plasma insulin [15]. Thus, *SLC35F3* was associated with MetS risk factors such as blood pressure, insulin, and visceral fat.

*SLC35F3* is also involved in carbohydrate metabolism [16,17,18]. *SLC35F3* transports thiamine into the cell, a coenzyme of pyruvate dehydrogenase [16]. The pyruvate dehydrogenase complex acts as a central regulator of the tricarboxylic acid cycle, a determinant step in carbohydrate metabolism [17,18]. Thiamine deficiency reduces pyruvate dehydrogenase activity and decreases oxidation capacity [17]. In subjects with the rs17514104 TT genotype of *SLC35F3*, erythrocyte thiamine content was decreased, and hereditary cardiovascular characteristics associated with thiamine deficiency were predicted [19]. This is because people with this genotype exhibit changes in cardiac output and systemic vascular resistance, which are consistent with people with thiamine deficiency, who also experience both cardiovascular and neurological signs [19]. Elevated blood pressure has been linked to *SLC35F3* variations associated with carbohydrate intake. *SLC35F3* has been linked to hypertension risk and protein-caloric malnutrition, thereby reflecting the coenzyme function of thiamine in carbohydrate metabolism [20]. This suggests that the *SLC35F3* may affect MetS through its role in carbohydrate metabolism.

Several studies have reported a link between carbohydrates and MetS, or between *SLC35F3* and MetS components such as hypertension. However, few studies have investigated the association between carbohydrates, MetS, and *SLC35F3*. Moreover, few studies have used a prospective cohort study design to elucidate the association between variations in this gene and the incidence of MetS associated with carbohydrate intake in Korean adults. Therefore, the present study aimed to examine the effects of the interaction between *SLC35F3* and carbohydrate consumption on the incidence of MetS in middle-aged Koreans.

## 2. Materials and Methods

### 2.1. Data Source and Study Participants

This prospective cohort study, which was based on Ansan-Ansung cohort data from the Korean Genome and Epidemiology Study (KoGES), utilized the Korean Association Resource Consortium (KARE) data. KoGES is a cohort study conducted to identify gene–environment interactions in chronic diseases common to Koreans [21]. Beginning in 2001, men and women between the ages of 40 and 69 who resided in Ansan and Ansung were recruited, and repeated examinations were conducted by recontacting the individuals every 2 years, with the 6th follow-up in 2013–2014. This study used data from the baseline to the 6th follow-up examination (2013–2014).

**Figure 1:**
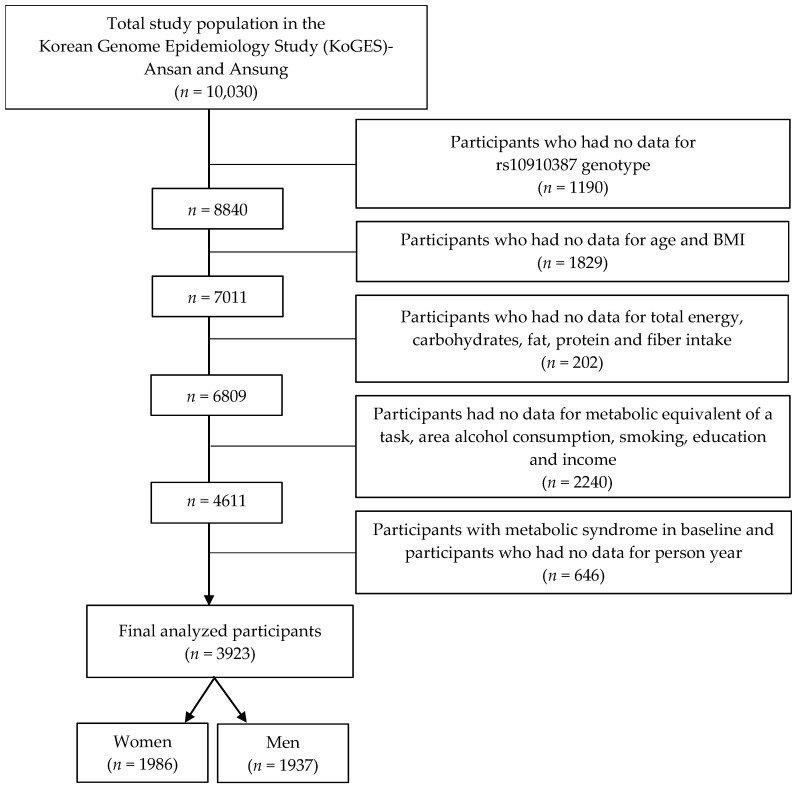
Flow chart for the study design: participants and exclusion criteria.

Out of a total of 10,030 participants, individuals were excluded according to the following criteria: individuals without rs10910387 genotyping data (*n* = 1190); people without age and body mass index (BMI) data (*n* = 1829); people without data on total energy, carbohydrate, fat, protein, and fiber intake (*n* = 202); people without data on metabolic equivalent of a task (MET), area, drinking status, smoking, education, and income (*n* = 2240); people with MetS in baseline and people without data on person-years (*n* = 646) (Figure 1). Ultimately, 3923 participants (1986 men and 1937 women) were included in the study.

### 2.2. Dietary Assessment

A baseline semi-quantitative food-frequency questionnaire (FFQ) was used to collect diet information. This FFQ presents 106 foods commonly consumed by Koreans, along with the reference amount, to investigate the yearly average consumption frequency and intake. This investigation was conducted under the assumption that eating habits do not change significantly. The intake frequency of food items was surveyed at a total of nine stages: almost no eating; once per month; 2–3 times per month; 1–2 times per week; 3–4 times per week; 5–6 times per week; once, twice, and thrice per day. The amount of intake for each food was assessed in three classifications: large, medium, and small. Responses were provided through observation of a picture of the presented amount. However, if there was no response to all FFQ items, there was no response to more than 12 FFQ items, there was no response to all rice items, or there was a high frequency of non-response, it was excluded from FFQ subjects. Additionally, a total energy intake per day lower than 100 kcal or greater than 10,000 kcal was removed from the FFQ analysis as abnormal energy intake. When calculating carbohydrate intake, foods that contribute to carbohydrates reaching 90% were selected, therefore, rice, barley, multigrain rice, rice cakes, ramen, noodles, dumplings, bread, pizza, hamburgers, cookies, potatoes, and sweet potatoes were used to obtain carbohydrate intake among 106 food groups in the FFQ.

Carbohydrate intake was differentially calculated to analyze HR and beta values. For the HR analysis, carbohydrate intake (g/day) was converted into % energy from carbohydrates. This was calculated by multiplying carbohydrate consumption (g/day) by four, dividing total energy intake (kcal/day), and multiplying by 100.

For beta value analysis, carbohydrate intake (g/day) was converted to carbohydrate intake g per 1000 kcal. This was calculated by dividing the carbohydrate intake (g/day) by the total energy intake (kcal/day) and multiplying by 1000.

### 2.3. Genotyping and Imputation

Based on the KARE genotyping data, single-nucleotide polymorphism (SNP) genotypes were chosen for this investigation. Affymetrix Genome-Wide Human SNP Array 5.0 (Affymetrix, Santa Clara, CA, USA) was used to genotype DNA samples. The exclusion criteria from analysis were as follows: genotype reading accuracy of less than 98%, high rates of missed genotype calls (4% or more), heterozygosity greater than 30%; or gender mismatch [22,23,24]. After genotyping, a GWAS was performed to select SNPs related to MetS. The relationship between each SNP and MetS incidence was assessed using multivariate logistic regression analysis adjusted for confounding variables.

**Figure 2:**
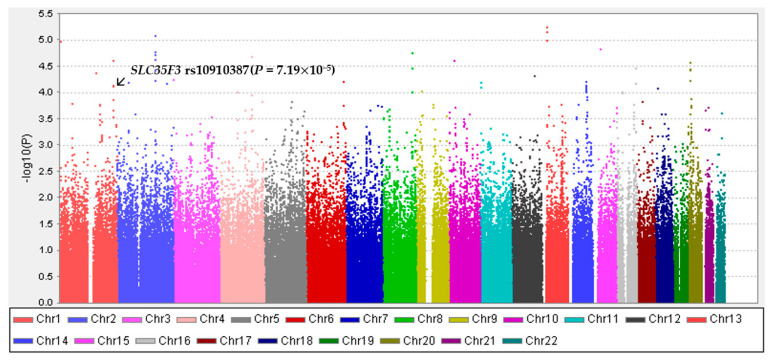
Manhattan plots for genome-wide association study (GWAS) of metabolic syndrome.

Rs10910387 of *SLC35F3* with a *p*-value of 7.19 × 10^−5^ was selected (Figure 2). Of the 352,228 SNPs obtained, 8840 corresponding to rs10910387 were used in this study, and an additive model for rs10910387 was used. The rs10910387 CC genotype was divided into major genotypes, and the TC and TT genotypes were divided into minor genotypes.

### 2.4. Assessment of Metabolic Syndrome (MetS)

MetS was defined as the presence of three or more of the five following five components above the diagnostic threshold: waist circumference, blood pressure, blood glucose, triglycerides, and HDL-cholesterol. Participants with MetS were excluded from the analysis at baseline. MetS was evaluated from the second to the sixth follow-up. Clinical figures for the diagnosis of MetS were derived from the NCEP-ATP III and KSSO recommendations [25].

Abdominal obesity was considered as a waist measurement ≥90 cm for men and ≥80 cm for women. Waist circumference was measured horizontally between the iliac ridge and rib without the clothes except for light inner wear [26]. A systolic blood pressure of at least 130 mmHg, diastolic blood pressure of at least 85 mmHg, history of hypertension, use of antihypertensive drugs, or ongoing hypertension treatment were all considered elevated blood pressure. Blood pressure was measured at least twice per visit while supporting the arms and back and measuring the arms at the heart level. Elevated fasting blood glucose was defined if the fasting blood glucose exceeds 100 mg/dL, diagnosed with diabetes, ongoing diabetes treatment, insulin treatment, or oral diabetes medication [27]. Triglyceride levels above 150 mg/dL were defined as hypertriglyceridemia [28]. Hypo HDL-cholesterolemia was defined as HDL cholesterol levels of <40 mg/dL for men and 50 mg/dL for women [2]. Triglyceride, HDL-C, and fasting blood glucose levels were assessed using ADVIA 1650 [24].

### 2.5. Assessment of Other Variables

The study was conducted by correcting variables such as age, sex, region, smoking and drinking status, BMI, education, income, and MET. Categorical variables were sex (men/women), region (Ansan/Ansung), smoking (none/past/current), drinking (none/past/current), education (elementary—technical college/university/graduate school), and income (<1, 1–3, >3 million won/month).

Age, BMI, MET score, and total energy intake were continuous variables. BMI was measured using Inbody 3.0 (Biospace). Physical activity was measured based on MET, and the average MET value for each physical activity was calculated by distributing each MET value based on the amount of physical activity. The average MET value was multiplied by the conversion of activity to hours, and the sum of all values was used to calculate MET hours per day, which was then multiplied by seven to obtain MET hours per week.

### 2.6. Statistical Analyses

To compare the general characteristics of the participants depending on the presence of MetS and the genotype of *SLC35F3* rs10910387, continuous variables were represented as mean ± standard deviation (SD) using the *t*-test and general linear model, and categorical variables were represented as frequency (%) using the chi-square test. PLINK 1.9 software (https://www.cog-genomics.org/plink/1.9, accessed on 20 October 2020) was used for genetic analysis. Carbohydrate intake in men and women was divided into tertiles to analyze the relationship between carbohydrate intake and MetS and the interaction between carbohydrates and genes on MetS and its elements (median (% energy from carbohydrates): men 63.4/69.5/75.3, women 64.9/71.6/77.4). MetS and its elements were set as dependent variables, and the genotype of *SLC35F3* rs10910387 and carbohydrate intake were set as independent variables.

A multivariate Cox proportional hazards model was used to calculate the HR and 95% CI for the following associations: carbohydrate intake and MetS; genetic variants and MetS; gene variants and MetS and its elements based on carbohydrate. The relationship between gene and MetS components and between carbohydrate intake and MetS components was estimated using multiple linear regression analysis for beta values and standard deviations. For the analysis, age, area, drinking and smoking status, BMI, total energy consumption, level of education, income, and MET were adjusted. SAS 9.4 (SAS Institute, Cary, NC, USA) was used for each statistical analysis; the results were considered significant when the *p*-value was less than 0.05.

## 3. Results

### 3.1. General Characteristics of the Study Participants Based on the Presence of Metabolic Syndrome

Table 1 shows the general characteristics of the study participants according to the presence or absence of MetS. The proportion of rs10910387 genotypes differed significantly only in men (*p* < 0.05). The normal group had a higher proportion of CC genotypes than the MetS group, whereas the MetS group had a higher proportion of TC and TT genotypes. Women in the MetS group were older than those in the normal group (*p* < 0.05). BMI and MetS risk factors, such as triglyceride levels, systolic and diastolic blood pressure, waist circumference, and fasting blood glucose values, were significantly higher in the MetS group than in the normal group in both men and women, whereas HDL-cholesterol levels were significantly lower (all, *p* < 0.05). In women, dietary intake differed significantly between the two groups: the MetS group showed a higher daily average consumption of protein and fiber, and a lower daily average fat intake (all, *p* < 0.05). Women with MetS also showed higher MET levels (*p* < 0.05). Ansan (urban) had a higher proportion of men and women than Ansung (rural) in both of the groups (*p* < 0.05). Furthermore, education and income levels differed significantly only in women. In terms of education level, both the MetS and normal groups had significantly higher rates of elementary/technical college than the rate of more than university. In the case of income level, the rate of 1–3 million won per month was much higher than the rate of ≤1 or ≥3 million won per month (all, *p* < 0.05). In men, there were significant differences in smoking status, and in both the MetS and normal groups, the current smoking rate was considerably greater than the past/non-smoking rates (all, *p* < 0.05).

### 3.2. General Characteristics of the Study Participants Based on Genetic Variation

The general characteristics of the study participants based on the *SLC35F3* rs10910387 genotype are shown in Table 2. Significant differences in the general characteristics according to genotype were found only in men. In men, the rs10910387 TC genotype had significantly higher diastolic blood pressure than the CC and TT genotypes (*p* < 0.05). The other variables did not differ significantly between the groups. Additionally, no significant differences were observed among the women.

### 3.3. Association of SLC35F3 rs10910387 with Metabolic Syndrome Components

Table 3 shows the GWAS results for the association between *SLC35F3* rs10910387 and MetS components. The analysis was performed using an additive model of the gene. This relationship was investigated using linear regression analysis, while controlling for age, region, and sex. The rs10910387 minor allele was positively related with diastolic blood pressure and triglyceride levels (beta 0.46, *p* = 0.036; beta 5.54, *p* = 0.007). There were no significant associations with other metabolic syndrome components.

### 3.4. Metabolic Syndrome Incidence Depending on Genotypes of SLC35F3 rs10910387

Table 4 shows MetS incidence based on the rs10910387 genotype. In men, TC and TT genotypes of rs10910387 had a higher incidence of MetS. In a multivariate Cox proportional hazards model, men with the TC and TT genotypes had a 1.19- and 1.48-fold greater incidence of MetS, respectively, adjusting for confounding factors such as age, compared to men with the CC genotype (HR 1.19, 95% CI 1.02–1.39, *p* = 0.03; HR 1.48, 95% CI 1.07–2.04, *p* = 0.02). Among women, MetS incidence was shown to be higher in people with the TT genotype. MetS incidence was 2.15 times higher in women with the TT genotype than in women with the CC genotype in an adjusted multivariable Cox proportional hazard model (HR 2.15, 95% CI 1.44–3.21, *p* = 0.0002).

### 3.5. Association of Carbohydrate Intake with Metabolic Syndrome Components

The relationship between carbohydrate intake and MetS components is shown in Table 5. Triglycerides and HDL-C values were significantly associated with carbohydrate intake in men. As carbohydrate intake increased, triglyceride levels increased, and HDL-C levels decreased (beta 0.42, *p* = 0.005; beta −0.02, *p* = 0.043). In women, carbohydrate intake was significantly negatively related to HDL-C levels. As carbohydrate intake increased, HDL-C levels decreased (beta −0.04, *p* = 0.004).

### 3.6. Metabolic Syndrome Incidence Depending on Carbohydrate Intake

Table 6 shows the incidence of MetS according to the carbohydrate intake. In men, there was an inconsistent trend of decrease and then increase in the number of patients with MetS as carbohydrate intake increased. However, in women, a consistent pattern of increase in carbohydrate intake was observed. The association between carbohydrate intake and MetS was not significant in men (HR 0.98, 95% CI 0.80–1.19, *p* = 0.83). In women, there were no statistically significant findings (HR 0.95, 95% CI 0.77–1.17, *p* = 0.64).

### 3.7. Metabolic Syndrome Incidence Depending on the Genotype of rs10910387 by Carbohydrate Intake

Table 7 shows the effect of carbohydrate intake on the association between the rs10910387 genotype and MetS incidence. In both men and women, the effect of *SLC35F3* variants on MetS incidence varied with carbohydrate intake. MetS incidence was 1.88 times higher in men with the TT genotype and with the highest carbohydrate intake than in those with the CC genotype and lowest carbohydrate intake (HR 1.88, 95% CI 1.03–3.41). In women, compared to the group with the CC genotype and tertile 1 of carbohydrate intake, the groups with the TT genotype and tertile 2 and 3 of carbohydrate intake exhibited a 2.22- and 2.53-fold increase in MetS incidence, respectively (HR 2.22, 95% CI 1.12–4.42; HR 2.53, 95% CI 1.38–4.61, respectively).

### 3.8. Metabolic Syndrome Components Incidence Depending on the Genotype of rs10910387 by Carbohydrate Intake

Table 8 displays the HR and 95% CI for the association between *SLC35F3* rs10910387 genotype and MetS components related to carbohydrate intake. In the group with the lowest carbohydrate intake, men with the TT genotype had a higher incidence of low HDL-cholesterolemia than those with the CC genotype (HR 1.79, 95% CI 1.02–3.16). In the group with the lowest carbohydrate intake, women with the TC and TT genotypes were more likely to develop hyperglycemia than women with the CC genotype (HR 1.34, 95% CI 1.02–1.75; HR 1.93, 95% CI 1.04–3.57).

## 4. Discussion

In this study, the KoGES data was used to investigate the relationship between MetS and *SLC35F3* in relation to carbohydrate intake. The T allele of *SLC35F3* rs10910387 was a risk allele, increasing the incidence of MetS, and its components, such as triglyceride, and diastolic blood pressure levels. Although carbohydrate intake had no effect on MetS, it did affect triglyceride and HDL-C levels, both of which are MetS components. The effect of *SLC35F3* variants on MetS incidence was found to be dependent on dietary carbohydrate intake. Men and women with the TT genotype and high carbohydrate intake had a higher MetS incidence than those with the CC genotype and low carbohydrate intake.

In this study, the *SLC35F3* rs10910387 T allele is a risk allele, and people with this allele had an increased incidence of MetS, as well as high triglyceride and diastolic blood pressure levels, both of which are indicators of MetS. Therefore, this study supports the findings of previous research. Hypertension, a risk factor for MetS, has been linked with *SLC35F3* [14,19]. In one study, *SLC35F3* rs10910387 was found to be positively related to systolic blood pressure in Korean women, and the TC genotype of the SNP was positively related to diastolic blood pressure [14]. In a Chinese study, hypertensive individuals with the risk allele of *SLC35F3* had higher diastolic blood pressure than those with the major allele [13]. According to another study, the risk allele of *SLC35F3* predicted genetic cardiovascular characteristics with decreased vascular resistance [19]. *SLC35F3* was significantly associated with visceral fat and fasting plasma insulin in addition to blood pressure [15].

Carbohydrate intake was not found to be related to MetS in this study, but was significantly associated with triglycerides and HDL-C, both of which are MetS components. Previous research into the link between carbohydrate intake and MetS has yielded inconsistent results. Studies conducted in Korea have discovered a significant relationship between carbohydrate consumption and MetS [8,9,10,29]. In one study, those with high carbohydrate intake had an unbalanced intake of macronutrients and other nutrients because they consumed more grains such as rice and fewer side dishes, and the risk of MetS increased with carbohydrate intake [10,30]. In another study, more than 70% of the energy intake ratio from carbohydrates was linked to an increase in BMI, blood pressure, fasting blood glucose, LDL cholesterol, and triglycerides in Korean women [29]. Even after controlling for covariates, low HDL-cholesterol and diabetes risk were found to be more prevalent among women with higher carbohydrate consumption [29]. However, other studies found no relationship between MetS and carbohydrate intake. No association between carbohydrate intake and MetS was found in a cross-sectional study of American adults [31]. Studies in China and Japan also revealed no connection between rice intake and metabolic diseases such as MetS and cardiovascular disorders [32,33]. Among elderly men living in Yangpyeong, no significant relationship was found between MetS elements and carbohydrate-related blood glucose values [34,35]. The carbohydrate glycemic index and load showed no association with HDL-C level, waist circumference, triglycerides, and fasting blood glucose [34]. Previous studies, including this study, would have been inconsistent with the results because the participants had different races and regions of residence, and, thus, different sources of carbohydrate intake. Furthermore, differences were observed based on the carbohydrate intake calculation method and dietary factors examined. Studies in Korea that found significant results mostly analyzed the association with MetS using the energy percentage of carbohydrates, whereas studies that did not show significant results were analyzed using carbohydrate intake (g/day) and carbohydrate-related dietary factors rather than a nutrient called carbohydrate. These studies indicate that it is difficult to conclude whether a single carbohydrate nutrient is directly related to MetS [36]. A type of carbohydrate, dietary fiber intake, was inversely associated with the risk of MetS [37,38]. Dietary fiber is carbohydrate that resists digestion and absorption in the small intestine with full or partial fermentation in the large intestine [39]. Dietary fiber reduces appetite and energy intake by inducing a feeling of satiety, and phytochemicals found in foods rich in dietary fiber help prevent MetS by suppressing oxidative stress and inflammation [40,41,42].

The carbohydrate quality index (CQI) is defined by the combination of several single components into a composite index, because a single nutrient cannot adequately capture the total quality of carbohydrate nutrition [43,44]. This index is an indicator of dietary carbohydrate quality, including the intake of whole grains, total fiber, glycemic index, and solid and liquid carbohydrates [45]. A previous study found an inverse relationship between the CQI and MetS [46]. In other words, MetS can be affected by the quality of carbohydrates as well as the amount of carbohydrates.

The present study showed that individuals with the TT genotype of *SLC35F3* rs10910387 were more likely to develop incidence of MetS when consuming a high-carbohydrate diet. In the analysis with MetS components, men with the TT genotype and the lowest carbohydrate intake were more likely to develop hypo HDL-cholesterolemia than men with the CC genotype and the lowest carbohydrate intake. Women with the TC and TT genotypes and the lowest carbohydrate intake had an increased incidence of hyperglycemia. This demonstrated that the *SLC35F3* rs10910387 T allele is a risk allele associated with MetS and its components, thereby clarifying the effect of *SLC35F3* and carbohydrate intake interactions on MetS. *SLC35F3* mediates the transport of thiamine, which acts as a coenzyme in carbohydrate metabolism in cells with increased energy requirements [47]. However, the *SLC35F3* risk allele (T allele) reduces the blood thiamine content [19]. In a previous study, the *SLC35F3* risk allele was found to be associated with inherited cardiovascular traits related to thiamine deficiency [19]. The cardiovascular characteristics induced by this allele include changes in heart rate, systemic vascular resistance, and pressure response, as well as impaired insulin synthesis and secretion and increased metabolic dysfunction [48,49]. In patients with diabetes, plasma thiamine levels were up to 76% lower than those in the control group, and thiamine-dependent enzymatic activity was found to be decreased in diabetic patients [50,51]. This showed that the risk allele of *SLC35F3* decreases thiamine, which is related to MetS such as cardiovascular disease and diabetes. In previous studies, thiamine deficiency is particularly dangerous in high-carbohydrate diets as it increased the metabolic requirements for thiamine [52]. However, a high-carbohydrate diet also lowers thiamine levels [53]. Adults who consumed high carbohydrates extracted from refined sugar and rice and had an unbalanced diet that consumed high carbohydrates were more likely to develop thiamine deficiency [54]. According to a previous study, when more than 55% of the total caloric intake was carbohydrate, the thiamine concentration decreased, and as carbohydrate intake increased, the thiamine concentration decreased further [53]. Thiamine deficiency caused by high carbohydrate intake is common in MetS and causes hypertension, hyperglycemia, and insulin resistance [55,56,57]. That is, a high-carbohydrate diet is metabolically linked to cardiovascular and metabolic illnesses by interacting with *SLC35F3* in connection with thiamine [58]. In summary, a high-carbohydrate diet and *SLC35F3* genetic variants induced thiamin deficiency, which may lead to the incidence of MetS.

This study has several strengths. First, it is the first study to identify how the interaction of *SLC35F3* with carbohydrates affects MetS in the Korean population. Additionally, this study has the distinction of being the first to use a prospective study design to identify the link between *SLC35F3* and MetS. Finally, as the study’s confounding variables, including age and BMI, were adjusted for in the analysis, it was possible to identify an independent impact of the interaction between carbohydrates and the *SLC35F3* gene variants on MetS. However, this study has some limitations. First, because this was a prospective cohort study, it was not possible to determine the mechanism by which the interaction between carbohydrates and genes affects MetS. Additionally, because the study was conducted on a specific population residing in Ansan and Ansung, generalizing the results to the entire Korean population is difficult. Finally, as the participants in this study were middle-aged and generally consumed large amount of carbohydrates, the carbohydrate intake variations of the participants were not large.

In conclusion, the risk allele of *SLC35F3* rs10910387 increased the incidence of MetS. The TC and TT genotypes of *SLC35F3* rs10910387 increased the incidence of MetS in men, and the TT genotype increased the incidence of MetS in women. Furthermore, high carbohydrate intake strengthened the link between *SLC35F3* rs10910387 minor allele and MetS in middle-aged Koreans. The incidence of MetS was further increased when both men and women had the rs10910387 TT genotype and were on a high-carbohydrate diet. This suggests that a genetic predisposition may synergistically interact with dietary carbohydrates to determine MetS risk in the future. It is difficult to conclude that the intake amount of a single nutrient called carbohydrates is directly related to MetS, and Koreans consume carbohydrates through various sources. In the future, it will be necessary to study the effect of the interaction between the quality of carbohydrate intake and genetic variation on MetS, considering both carbohydrate intake and carbohydrate intake from various sources.

## Figures and Tables

**Table 1:** General characteristics of the study participants based on the presence of metabolic syndrome.

Variables	Men		*p* Value	Women		*p* Value
	Metabolic Syndrome (*n* = 747)	No Metabolic Syndrome (*n* = 1239)		Metabolic Syndrome (*n* = 706)	No Metabolic Syndrome (*n* = 1231)	
rs10910387			0.0002			0.05
CC	470 (62.9%)	864 (69.7%)		479 (67.9%)	870 (70.7%)	
TC	236 (31.6%)	344 (27.8%)		201 (28.5%)	337 (27.4%)	
TT	41 (5.5%)	31 (2.5%)		26 (3.7%)	24 (2.0%)	
Age (years)	50.4 ± 8.2	50.1 ± 8.6	0.47	52.7 ± 8.6	48.2 ± 7.7	<0.0001
BMI (kg/m^2^)	24.7 ± 2.4	23.1 ± 2.5	<0.0001	25.1 ± 2.9	23.4 ± 2.7	<0.0001
Waist circumference (cm)	84.5 ± 5.8	79.7 ± 6.4	<0.0001	81.2 ± 7.5	75.2 ± 7.5	<0.0001
Blood pressure						
Systolic blood pressure (mmHg)	121.5 ± 16.3	115.9 ± 15.0	<0.0001	120.0 ± 16.6	109.5 ± 14.9	<0.0001
Diastolic blood pressure (mmHg)	82.0 ± 10.5	78.1 ± 10.2	<0.0001	78.4 ± 9.9	72.3 ± 9.7	<0.0001
Triglycerides (mg/dL)	181.3 ± 128.4	132.7 ± 65.6	<0.0001	133.2 ± 67.3	109.8 ± 49.0	<0.0001
Glucose (mg/dL)	89.9 ± 19.8	85.6 ± 15.3	<0.0001	83.9 ± 15.4	80.1 ± 9.7	<0.0001
HDL-cholesterol (mg/dL)	42.6 ± 8.4	47.0 ± 10.0	<0.0001	45.9 ± 9.1	49.9 ± 10.4	<0.0001
Dietary intake						
Calorie intake (kcal)	2038.7 ± 697.2	2013.6 ± 594.6	0.41	1901.9 ± 674.6	1881.8 ± 682.4	0.53
Carbohydrate intake (g)	71.5 ± 30.2	69.7 ± 26.2	0.18	64.1 ± 27.0	65.4 ± 32.7	0.34
Fat intake (g)	37.3 ± 23.2	36.6 ± 18.8	0.48	29.4 ± 17.3	32.3 ± 22.2	0.002
Protein intake (g)	348.7 ± 109.8	345.7 ± 97.7	0.54	340.8 ± 123.3	328.4 ± 108.1	0.03
Fiber intake (g)	7.0 ± 3.6	6.8 ± 3.0	0.11	7.2 ± 3.8	6.9 ± 3.3	0.04
MET (hours/week)	166.1 ± 102.4	159.2 ± 97.3	0.13	163.4 ± 102.3	143.0 ± 82.9	<0.0001
Area			0.004			<0.0001
Ansung	241 (32.3%)	325 (26.2%)		329 (46.6%)	295 (24.0%)	
Ansan	506 (67.7%)	914 (73.8%)		377 (53.4%)	936 (76.0%)	
Education			0.07			0.007
Elementary/technical college	611 (81.8%)	1017 (82.1%)		680 (96.3%)	1143 (92.9%)	
University	119 (15.9%)	173 (14.0%)		23 (3.3%)	81 (6.6%)	
Graduate school	17 (2.3%)	49 (4.0%)		3 (0.4%)	7 (0.6%)	
Income (million won/month)			0.63			<0.0001
<1	160 (21.4%)	268 (21.6%)		281 (39.8%)	282 (22.9%)	
1–3	381 (51.0%)	653 (52.7%)		326 (46.2%)	669 (54.4%)	
>3	206 (27.6%)	318 (25.7%)		99 (14.0%)	280 (22.8%)	
Smoking			0.004			0.49
None	132 (17.7%)	285 (23.0%)		675 (95.6%)	1189 (96.6%)	
Past	227 (30.4%)	394 (31.8%)		8 (1.1%)	13 (1.1%)	
Current	388 (51.9%)	560 (45.2%)		23 (3.3%)	29 (2.4%)	
Drinking			0.16			0.61
None	129 (17.3%)	237 (19.1%)		470 (66.6%)	837 (68.0%)	
Past	57 (7.6%)	118 (9.5%)		24 (3.4%)	33 (2.7%)	
Current	561 (75.1%)	884 (71.4%)		212 (30.0%)	361 (29.3%)	

Categorical variables are represented as number (%). Continuous variables are represented as mean ± SD. The *p* values were based on the *t*-test for continuous variables and the chi-square test for categorical variables. Abbreviations: BMI—Body mass index; HDL—High-density lipoprotein; MET—Metabolic equivalent of task.

**Table 2:** General characteristics of the study participants based on the *SLC35F3* rs10910387 genotype.

Variables	Men			*p* Value	Women			*p* Value
	CC (*n* = 1334)	TC (*n* = 580)	TT (*n* = 72)		CC (*n* = 1349)	TC (*n* = 538)	TT (*n* = 50)	
Age (years)	50.0 ± 8.3	50.6 ± 8.7	50.5 ± 9.3	0.42	49.9 ± 8.4	49.7 ± 8.1	48.1 ± 7.8	0.27
BMI (kg/m^2^)	23.6 ± 2.6	23.8 ± 2.6	24.3 ± 2.4	0.06	24.0 ± 2.8	24.0 ± 3.0	24.0 ± 2.8	0.98
Waist circumference (cm)	81.4 ± 6.7	81.8 ± 6.4	81.7 ± 6.4	0.39	77.3 ± 7.9	77.6 ± 8.3	77.1 ± 8.6	0.73
Blood pressure								
Systolic blood pressure (mmHg)	117.5 ± 15.5	119.0 ± 15.8	119.6 ± 18.9	0.09	113.2 ± 16.0	113.7 ± 17.0	112.2 ± 16.8	0.75
Diastolic blood pressure (mmHg)	79.2 ± 10.5	80.5 ± 10.5	79.2 ± 10.3	0.04	74.5 ± 10.1	74.5 ± 10.6	75.1 ± 9.7	0.92
Triglycerides (mg/dL)	148.0 ± 91.7	156.8 ± 109.4	158.5 ± 86.6	0.15	118.1 ± 56.9	119.5 ± 60.5	112.7 ± 37.0	0.7
Glucose (mg/dL)	87.5 ± 18.1	86.6 ± 15.1	87.3 ± 17.1	0.61	81.5 ± 12.3	81.3 ± 12.2	81.1 ± 7.3	0.92
HDL-cholesterol (mg/dL)	45.6 ± 9.6	44.8 ± 9.7	45.5 ± 10.6	0.23	48.4 ± 10.2	48.6 ± 10.0	47.7 ± 9.7	0.76
Dietary intake								
Calorie intake (kcal)	2018.2 ± 649.8	2035.0 ± 608.9	2018.7 ± 578.7	0.87	1889.0 ± 669.8	1903.8 ± 716.4	1735.6 ± 496.1	0.25
Protein intake (g)	70.1 ± 28.0	71.1 ± 27.7	69.3 ± 24.0	0.75	64.7 ± 29.4	65.9 ± 34.5	61.4 ± 22.9	0.54
Fat intake (g)	36.8 ± 20.9	37.3 ± 20.2	36.0 ± 16.5	0.81	31.1 ± 19.6	31.7 ± 23.1	29.4 ± 18.4	0.7
Carbohydrate intake (g)	346.1 ± 104.4	348.4 ± 98.8	348.8 ± 94.8	0.89	333.3 ± 113.1	334.8 ± 118.2	302.5 ± 85.7	0.16
Fiber intake (g)	6.8 ± 3.2	7.0 ± 3.3	6.8 ± 2.8	0.35	6.9 ± 3.4	7.2 ± 3.8	6.4 ± 2.9	0.3
MET (hours/week)	163.2 ± 99.0	161.1 ± 101.3	142.4 ± 86.6	0.22	149.7 ± 90.6	149.8 ± 91.9	178.8 ± 86.8	0.08
Area				0.29				0.67
Ansung	379 (28.4%)	172 (29.7%)	15 (20.8%)		434 (32.2%)	171 (31.8%)	19 (38.0%)	
Ansan	955 (71.6%)	408 (70.3%)	57 (79.2%)		915 (67.8%)	367 (68.2%)	31 (62.0%)	
Education				0.24				0.47
Elementary/technical college	1082 (81.1%)	483 (83.3%)	63 (87.5%)		1267 (93.9%)	506 (94.1%)	50 (100.0%)	
University	208 (15.6%)	75 (12.9%)	9 (12.5%)		74 (5.5%)	30 (5.6%)	0 (0%)	
Graduate school	44 (3.3%)	22 (3.8%)	0 (0%)		8 (0.6%)	2 (0.4%)	0 (0%)	
Income (million won/month)				0.78				0.43
<1	282 (21.1%)	130 (22.4%)	16 (22.2%)		388 (28.8%)	161 (29.9%)	14 (28.0%)	
1–3	688 (51.6%)	308 (53.1%)	38 (52.8%)		691 (51.2%)	282 (52.4%)	22 (44.0%)	
>3	364 (27.3%)	142 (24.5%)	18 (25.0%)		270 (20.0%)	95 (17.7%)	14 (28.0%)	
Smoking				0.7				0.32
None	281 (21.1%)	118 (20.3%)	18 (25.0%)		1299 (96.3%)	519 (96.5%)	46 (92.0%)	
Past	410 (30.7%)	192 (33.1%)	19 (26.4%)		12 (0.9%)	8 (1.5%)	1 (2.0%)	
Current	643 (48.2%)	270 (46.6%)	35 (48.6%)		38 (2.8%)	11 (2.0%)	3 (6.0%)	
Drinking				0.81				0.61
None	243 (18.2%)	106 (18.3%)	17 (23.6%)		907 (67.2%)	367 (68.2%)	33 (66.0%)	
Past	120 (9.0%)	50 (8.6%)	5 (6.9%)		36 (2.7%)	18 (3.4%)	3 (6.0%)	
Current	971 (72.8%)	424 (73.1%)	50 (69.4%)		406 (30.1%)	153 (28.4%)	14 (28.0%)	

Continuous variables are represented as mean ± SD. Categorical variables are represented as a number (%). The *p* values were based on the general linear model for continuous variables and the chi-square test for categorical variables. Abbreviations: BMI—Body mass index: HDL—High-density lipoprotein; MET—Metabolic equivalent of task.

**Table 3:** Association of *SLC35F3* rs10910387 with metabolic syndrome components.

SNP	rs10910387 (*SLC35F3*)	
Minor Allele: T	Beta ± SE	Add *p*
Waist circumference (cm)	0.23 ± 0.17	0.161
Systolic blood pressure (mmHg)	0.38 ± 1.11	0.266
Diastolic blood pressure (mmHg)	0.46 ± 2.10	0.036
Triglycerides (mg/dL)	5.54 ± 2.68	0.007
Glucose (mg/dL)	0.12 ± 0.27	0.784
HDL-cholesterol (mg/dL)	−0.30 ± −1.53	0.126

Abbreviations: SE—Standard Error; Add—Additive model; HDL—High-Density Lipoprotein. Adjusted for age, area, and sex.

**Table 4:** Adjusted hazard ratios (HRs) and 95% confidence intervals (CIs) of metabolic syndrome incidence depending on the genotype of *SLC35F3* rs10910387.

**Men**		**Metabolic Syndrome**	
**rs10910387 (*SLC35F3*)**	**Cases/Total**	**HR (95% CI)**	***p* Value**
CC	470/1334	1.00 (Ref)	
TC	236/580	1.19 (1.02–1.39)	0.03
TT	41/72	1.48 (1.07–2.04)	0.02
**Women**		**Metabolic Syndrome**	
**rs10910387 (*SLC35F3*)**	**Cases/Total**	**HR (95% CI)**	***p* Value**
CC	479/1349	1.00 (Ref)	
TC	201/538	1.11 (0.94–1.31)	0.22
TT	26/50	2.15 (1.44–3.21)	0.0002

Abbreviations: HR—Hazard Ratio; CI—Confidence Interval. Adjusted for age (years, continuous), area (Ansung, Ansan), BMI (kg/m^2^, continuous), smoking (none, past, current), drinking status (none, past, current), education (elementary—technical college, university, grade school), income (<1, 1–3, >3 million won/month), total energy intake (%, continuous), and metabolic equivalent of a task (continuous).

**Table 5:** Association of carbohydrate intake (g/1000 kcal per day) with metabolic syndrome components.

	Men (*n* = 1986)		Women (*n* = 1937)	
	Beta ± SE	*p* Value	Beta ± SE	*p* Value
Waist circumference (cm)	−0.01 ± 0.01	0.082	0.01 ± 0.01	0.310
Systolic blood pressure (mmHg)	0.01 ± 0.02	0.648	−0.003 ± 0.02	0.896
Diastolic blood pressure (mmHg)	−0.01 ± 0.02	0.739	0.01 ± 0.01	0.375
Triglycerides (mg/dL)	0.42 ± 0.15	0.005	0.09 ± 0.09	0.284
Fasting blood glucose (mg/dL)	−0.04 ± 0.03	0.113	−0.003 ± 0.02	0.863
HDL-cholesterol (mg/dL)	−0.02 ± 0.01	0.043	−0.04 ± 0.02	0.004

Abbreviations: SE—Standard Error; CI—Confidence Interval; HDL—High-Density Lipoprotein. Adjusted for age (years, continuous), area (Ansung, Ansan), BMI (kg/m^2^, continuous), smoking (none, past, current), drinking status (none, past, current), education (elementary—technical college, university, grade school), income (<1, 1–3, >3 million won/month), total energy intake (%, continuous), and metabolic equivalent of a task (continuous).

**Table 6:** Adjusted hazard ratios (HRs) and 95% confidence intervals (CIs) of metabolic syndrome incidence depending on carbohydrate intake.

**Men**		**Metabolic Syndrome**	
**Carbohydrate (% Energy)**	**Cases/Total**	**HR (95% CI)**	***p* Value**
Tertile 1	258/662	1.00 (Ref)	
Tertile 2	231/662	0.87 (0.73–1.05)	0.14
Tertile 3	258/662	0.98 (0.80–1.19)	0.83
**Women**		**Metabolic Syndrome**	
**Carbohydrate (% Energy)**	**Cases/Total**	**HR (95% CI)**	***p* Value**
Tertile 1	191/645	1.00 (Ref)	
Tertile 2	233/646	1.07 (0.86–1.30)	0.53
Tertile 3	282/646	0.95 (0.77–1.17)	0.64

Abbreviations: HR—Hazard Ratio; CI—Confidence Interval. Adjusted for age (years, continuous), area (Ansung, Ansan), BMI (kg/m^2^, continuous), smoking (none, past, current), drinking status (none, past, current), education (elementary—technical college, university, grade school), income (<1, 1–3, >3 million won/month), total energy intake (%, continuous), and metabolic equivalent of a task (continuous).

**Table 7:** Adjusted hazard ratios (HRs) and 95% confidence intervals (CIs) of metabolic syndrome incidence depending on the genotype of *SLC35F3* rs10910387 by carbohydrate intake.

**Men**	**Carbohydrate (% Energy)**				**Women**	**Carbohydrate (% Energy)**			
	**Tertile 1**	**Tertile 2**	**Tertile 3**			**Tertile 1**	**Tertile 2**	**Tertile 3**	
Median	63.4	69.5	75.3		Median	64.9	71.6	77.4	
Person-years	5599	5688	5434	16,721	Person-years	5809	5742	5235	16,786
Incident cases (*n*)	258/662	231/662	258/662	747/1986	Incident cases (*n*)	191/645	233/646	282/646	706/1937
**Adjusted HR (95% CI)**	**HR (95% CI)**	**HR (95% CI)**	**HR (95% CI)**	***p* interaction**	**Adjusted HR (95% CI)**	**HR (95% CI)**	**HR (95% CI)**	**HR (95% CI)**	***p* interaction**
CC	1.00 (Ref)	0.83 (0.66–1.04)	0.90 (0.71–1.14)	0.16	CC	1.00 (Ref)	1.01 (0.80–1.28)	0.95 (0.75–1.21)	0.79
TC	1.02 (0.77–1.34)	1.03 (0.78–1.36)	1.19 (0.90–1.57)		TC	1.11 (0.80–1.54)	1.26 (0.94–1.69)	0.97 (0.72–1.30)	
TT	1.48 (0.86–2.56)	1.02 (0.60–1.74)	1.88 (1.03–3.41)		TT	1.45 (0.59–3.57)	2.22 (1.12–4.42)	2.53 (1.38–4.61)	

Abbreviations: HR—Hazard Ratio; CI—Confidence Interval. Adjusted for age (years, continuous), area (Ansung, Ansan), BMI (kg/m^2^, continuous), smoking (none, past, current), drinking status (none, past, current), education (elementary—technical college, university, grade school), income (<1, 1–3, >3 million won/month), total energy intake (%, continuous), and metabolic equivalent of a task (continuous).

**Table 8:** Adjusted hazard ratios (HRs) and 95% confidence intervals (CIs) of metabolic syndrome components incidence depending on the genotype of *SLC35F3* rs10910387 by carbohydrate intake.

	Men					Women			
	Carbohydrate (% Energy)					Carbohydrate (% Energy)			
	Tertile 1	Tertile 2	Tertile 3			Tertile 1	Tertile 2	Tertile 3	
	HR (95% CI)	HR (95% CI)	HR (95% CI)	*p* Interaction		HR (95% CI)	HR (95% CI)	HR (95% CI)	*p* Interaction
Abdominal obesity				0.89	Abdominal obesity				0.21
CC	1.00 (Ref)	1.00 (0.78–1.29)	0.85 (0.65–1.11)		CC	1.00 (Ref)	0.97 (0.76–1.25)	1.04 (0.81–1.34)	
TC	1.10 (0.80–1.52)	1.21 (0.90–1.62)	0.96 (0.69–1.33)		TC	1.33 (0.95–1.86)	1.06 (0.77–1.46)	0.92 (0.66–1.27)	
TT	1.32 (0.64–2.71)	1.38 (0.79–2.40)	1.17 (0.63–2.17)		TT	0.70 (0.22–2.19)	1.72 (0.70–4.22)	1.00 (0.50–1.99)	
Elevated blood pressure				0.53	Elevated blood pressure				0.74
CC	1.00 (Ref)	0.82 (0.65–1.04)	0.90 (0.71–1.14)		CC	1.00 (Ref)	1.10 (0.87–1.41)	0.98 (0.77–1.27)	
TC	0.92 (0.68–1.24)	0.86 (0.64–1.16)	0.98 (0.73–1.32)		TC	1.02 (0.73–1.44)	1.21 (0.89–1.64)	1.01 (0.74–1.37)	
TT	0.89 (0.42–1.91)	0.93 (0.52–1.68)	0.79 (0.35–1.81)		TT	1.10 (0.48–2.50)	1.67 (0.80–3.47)	0.78 (0.32–1.91)	
Elevated fasting glucose				0.68	Elevated fasting glucose				0.17
CC	1.00 (Ref)	0.90 (0.74–1.08)	0.83 (0.68–1.02)		CC	1.00 (Ref)	0.96 (0.77–1.19)	0.87 (0.69–1.09)	
TC	1.05 (0.83–1.33)	0.92 (0.72–1.18)	1.03 (0.81–1.31)		TC	1.34 (1.02–1.75)	1.09 (0.83–1.43)	0.95 (0.72–1.25)	
TT	1.28 (0.71–2.29)	0.87 (0.50–1.49)	0.74 (0.36–1.52)		TT	1.93 (1.04–3.57)	0.92 (0.38–2.26)	1.03 (0.55–1.92)	
Elevated triglycerides				0.84	Elevated triglycerides				0.1
CC	1.00 (Ref)	0.95 (0.74–1.23)	1.06 (0.81–1.38)		CC	1.00 (Ref)	1.12 (0.89–1.41)	1.02 (0.80–1.30)	
TC	1.03 (0.74–1.42)	1.11 (0.80–1.55)	0.96 (0.68–1.37)		TC	0.80 (0.57–1.12)	1.19 (0.88–1.61)	1.26 (0.93–1.71)	
TT	1.35 (0.55–3.32)	0.87 (0.44–1.73)	1.59 (0.76–3.31)		TT	1.30 (0.57–2.96)	1.05 (0.42–2.60)	1.50 (0.80–2.79)	
Low HDL cholesterol				0.56	Low HDL cholesterol				0.76
CC	1.00 (Ref)	0.95 (0.77–1.19)	0.98 (0.79–1.23)		CC	1.00 (Ref)	0.99 (0.75–1.29)	1.01 (0.76–1.35)	
TC	0.81 (0.60–1.09)	1.11 (0.84–1.46)	1.10 (0.83–1.47)		TC	1.03 (0.72–1.48)	1.17 (0.83–1.67)	0.96 (0.67–1.37)	
TT	1.79 (1.02–3.16)	0.79 (0.42–1.51)	1.16 (0.60–2.29)		TT	1.01 (0.37–2.75)	1.32 (0.53–3.33)	1.96 (0.91–4.24)	

Abbreviations: HR—Hazard Ratio; CI—Confidence Interval. Adjusted for age (years, continuous), area (Ansung, Ansan), BMI (kg/m^2^, continuous), smoking (none, past, current), drinking status (none, past, current), education (elementary—technical college, university, grade school), income (<1, 1–3, >3 million won/month), total energy intake (%, continuous), and metabolic equivalent of a task (continuous).

## Data Availability

The data underlying the results of our study are not publicly available, because of the data policy of KoGES. Data are available from the Division of Genetic Epidemiology and Health Index, NIH, Korea Centers for Disease Control and Prevention, for researchers who meet the criteria for access to confidential data.
